# Transcriptional Reprogramming of Wheat and the Hemibiotrophic Pathogen *Septoria tritici* during Two Phases of the Compatible Interaction

**DOI:** 10.1371/journal.pone.0081606

**Published:** 2013-11-26

**Authors:** Fen Yang, Wanshun Li, Hans J. L. Jørgensen

**Affiliations:** 1 Department of Plant and Environmental Sciences, Faculty of Science, University of Copenhagen, Frederiksberg, Denmark; 2 BGI-Tech, BGI, Shenzhen, China; Seoul National University, Korea, Republic Of

## Abstract

The disease septoria leaf blotch of wheat, caused by fungal pathogen *Septoria tritici*, is of worldwide concern. The fungus exhibits a hemibiotrophic lifestyle, with a long symptomless, biotrophic phase followed by a sudden transition to necrotrophy associated with host necrosis. Little is known about the systematic interaction between fungal pathogenicity and host responses at specific growth stages and the factors triggering the transition. In order to gain some insights into global transcriptome alterations in both host and pathogen during the two phases of the compatible interaction, disease transition was monitored using pathogenesis-related gene markers and H_2_O_2_ signature prior to RNA-Seq. Transcriptome analysis revealed that the slow symptomless growth was accompanied by minor metabolic responses and slightly suppressed defences in the host, whereas necrotrophic growth was associated with enhanced host responses involving energy metabolism, transport, signalling, defence and oxidative stress as well as a decrease in photosynthesis. The fungus expresses distinct classes of stage-specific genes encoding potential effectors, probably first suppressing plant defence responses/facilitating the symptomless growth and later triggering life style transition and inducing host necrosis/facilitating the necrotrophic growth. Transport, signalling, anti-oxidative stress mechanisms and apoplastic nutrient acquisition play important roles in the entire infection process of *S. tritici*. Our findings uncover systematic *S. tritici*-induced expression profiles of wheat related to specific fungal infection strategies and provide a transcriptome resource for studying both hosts and pathogens in plant-Dothideomycete interactions.

## Introduction

*Septoria tritici* (teleomorph *Mycosphaerella graminicola*) is the causal agent of septoria leaf blotch, a foliar disease of wheat (*Triticum aestivum*) that poses a significant threat to global food production [1]. *S. tritici* penetrates host leaves through the stomata and grows slowly as filamentous hyphae in the intercellular spaces between the wheat mesophyll cells, typically up to 9–11 days [2,3]. Subsequently, the fungus exhibits a rapid switch to necrotrophy immediately prior to symptom expression at 12–20 days after penetration [3–5]. This transition is associated with induction of host defence responses that have been suggested to share characteristics with host programmed cell death (PCD) and differential regulation of signalling pathways [4,6]. The necrotrophic process involves cell-wall degradation and accumulation of H_2_O_2_, resulting in massive collapse of mesophyll tissue, leakage of nutrients from dying plant cells into the apoplastic spaces, rapid increase of fungal biomass and sporulation in characteristic necrotic foliar blotches [3,5,7,8]. The key features of this fungus, distinguishing it from most other current models, are a very long period of symptomless growth prior to a rapid transition to necrotrophy and that it remains extracellular with respect to host cells during the entire infection process without forming any specialised feeding structures [2,3,6]. However, the molecular basis underlying this transition remains unclear. This basis includes what the pathogen relies on to facilitate the long initial symptomless growth and subsequently trigger the transition of its lifestyle associated with host cell death and how the fungus copes with the activation of plant responses during the different infection phases. To address these issues, several investigations have attempted to understand *S. tritici* gene function, particularly the role of small secreted protein effectors. Likewise, fungal transcriptome profiles have been studied in adaption to environmental changes as well as host responses in cultivars with different susceptibility to infection. The research has been facilitated by the publication of the full genome sequence of *S. tritici*, revealing 10933 genes [9].

Effectors are believed to enable colonisation by the pathogen by interfering with a variety of plant-encoded virulence targets and to effectively suppress or activate defence responses. They can be recognised by disease resistance proteins, which subsequently trigger plant immunity [8]. In *S. tritici*, it has been suggested that the effectors may trigger disease transition, appearance of disease lesions and host cell death [10]. The only effector described in *S. tritici* is a LysM homologue shown to contribute to virulence in wheat [8]. Genes encoding secreted proteins with intragenic coding repeats within *S. tritici* populations were suggested to play potential roles as effectors [11]. *In silico* prediction of the secretome based on the full genome sequence identified 492 candidate virulence effectors and revealed a protein family encoding secreted (chloro)peroxidases, which is expanded within all family members of Mycosphaerellaceae [12]. Transcription profiling of *S. tritici in planta* using a microarray containing 2563 genes or expressed sequence tag (EST) libraries identified genes encoding cell-wall-degrading enzymes (CWDEs) and genes involved in signal transduction and transport [5] and revealed fungal physiological adaption with respect to membrane transport, chemical and oxidative stress mechanisms and metabolism during the rapid growth transition [4,13]. However, these transcriptome studies provide limited or ambiguous information on fungal pathogenicity based on a low number of identifications (150 genes in the biotrophic phase and max. 450 genes in the necrotrophic phase). 

Studies on host responses in the wheat-*S. tritici* interaction revealed that H_2_O_2_ is important in the defence of wheat against the pathogen. During a compatible interaction, H_2_O_2_ levels increased dramatically and peaked at the late necrotrophic stage, implying that this was likely a stress-response and not involved in defence [3,7]. Other responses reported include expression of a wheat protein disulfide isomerase gene and pathogenesis-related (PR) genes [14,15] as well as structural defence responses [15]. Biochemical investigations furthermore showed DNA laddering, translocation of cytochrome c from mitochondria to the cytosol, loss of host cell membrane integrity, degradation of host total RNA and differential regulation of host mitogen-activated protein kinase (MAPK) pathways during symptom development in a compatible interaction [4,6]. Recently, large-scale shotgun proteomics and phosphoproteomics have been conducted on *S. tritici*-infected wheat leaves from the symptomless stage, suggesting that resistance is likely related to rapidly and intensively triggered signal transduction cascades resulting in a multiple-level activation of transcription and translation processes of defence responses [16]. 

Transcriptomics is an important tool to investigate the regulatory mechanisms between the host and the pathogen as it can shed light on the shifts of metabolic and cellular processes due to the interaction. Among the various technologies of transcriptome profiling, RNA-Seq has become a revolutionary tool and has recently been employed in the study of plant-pathogen interactions. It can distinguish between paralogous genes, detect and quantify transcripts of low or high abundance and identify transcript sequence polymorphisms and novel trans-splicing and splice isoforms [17]. Moreover, there is no strict requirement for a reference genome sequence, which is required in hybridization-based approaches such as microarray. Although transcriptome studies have been carried out in *S. tritici in vitro* and *in planta*, limited information about fungal gene expression has been obtained, in particular during the initial symptomless stage, due to low fungal biomass hardly detected by previous technologies. In addition, lack of large-scale systematic studies of host responses to *S. tritici* at RNA level strongly encouraged us to investigate the host transcriptome responses to infection. 

Therefore, we used RNA-Seq in the present study to follow the transcriptional reprogramming with a key focus on both host and fungus simultaneously at distinct stages of the compatible interaction. These stages and disease transition were clearly defined by characterisation of fungal biomass, two essential plant PR genes/proteins (β-1,4-glucanase and chitinase) and ROS prior to transcriptome analysis. For the first time, we were not only able to study transcriptome changes (approx. 1800 transcripts) across the different growth phases in *S. tritici*, but also to obtain broad-spectrum insights into the host molecular responses corresponding to the distinct lifestyle phases of this pathogen. To our best knowledge, this is the first RNA-Seq study performed in a member of the Dothideomycetes. Our study has considerably expanded the knowledge obtained from previous gene expression investigations in *S. tritici*. 

## Materials and Methods

### Plant growth and inoculation

Growth of the susceptible wheat cv. Sevin, preparation of inoculum of *S. tritici* isolate IPO323 and inoculation were performed as described by Shetty et al. [3]. Control plants were mock-inoculated with water. Approximately 20 leaves were collected from two separate pots, serving as one biological replicate. Two biological replicates for inoculated and control samples were harvested every day from 3 to 14 days after inoculation (dai) and immediately frozen in liquid nitrogen. The leaf samples were ground in liquid nitrogen and stored at -80 °C until use. 

### Histochemical staining for H_2_O_2_ and visualization of fungal structures

*In vivo* detection of H_2_O_2_ was carried out using 3,3’-diaminobenzidine (DAB, Sigma) as described by Shetty et al. [3]. The leaves were then cleared, stained by 0.1% Evans blue in lactoglycerol to visualise the fungal surface structures and studied by light microscopy [3]. 

### Quantification of fungal biomass

Total DNA was extracted from infected and control samples using the DNeasy Plant Mini Kit (Qiagen, Venlo, The Netherlands). Fungal DNA was determined by qPCR using primers for *S. tritici* mating type gene 1-1 [18] with total DNA as template as previously described [19].

### Enzyme assay and western blotting

Water-soluble protein was extracted in 50 mM sodium acetate (pH 5.2) at 4 °C. Protein concentration in the extracts was determined by the Bio-Rad Protein Assay (Bio-Rad) with bovine serum albumin as standard. β-1,3-glucanase activity was assayed as described by Shetty et al. [15]. Briefly, protein samples were incubated with 0.1% (w/v) laminarin in 0.05 M sodium acetate buffer (pH 5.2) for 15 min at 37 °C. The reaction was stopped by adding DNS reagent containing 0.5% (w/v) 3,5-dinitrosalicylic acid (Sigma) and 15% (w/v) potassium sodium tartrate tetrahydrate (Sigma) followed by boiling for 10 min. The absorbance was measured in an ELISA-reader at 540 nm. A standard curve relating the amount of glucose equivalents to the absorbance at 540 nm was used to determine the activity. Chitinase activity was measured by the Chitinase Assay Kit (Sigma) following the manufacturer’s protocol. Five µg protein were separated on Criterion^TM^ XT Precast Gels (12% Bis-Tris, Bio-Rad) followed by blotting to nitrocellulose membranes (Whatman). Western blotting using rabbit antibodies against barley chitinase (kindly provided by David B. Collinge, University of Copenhagen) was performed as described by Yang et al. [20]. 

### RNA extraction and qPCR

RNA extraction, gDNA removal, cDNA synthesis and qPCR were performed as previously described [20,21]. Primers for genes encoding wheat β-1,3-glucanase and chitinase were used according to Shetty et al. [15]. The wheat 25S rRNA gene (Forward primer: 5’-AAGGCCGAAGAGGAGAAAGGT-3’; Reversed primer: 5’-CGTCCCTTAGGATCGGCTTAC-3’) served as reference gene. Relative expression of genes was determined using the formula: relative expression = 2^-(ΔCt target gene - ΔCt reference gene)^ where Ct refers to the threshold cycle in PCR [20]. All relative expression values of genes are reported as means ±SD. Analysis to reveal significant differences in gene expression in infected Sevin *versus* the respective controls at each time point was performed by Student’s *t*-test.

### Construction of cDNA library, data filtering and transcript assembly

Total RNA (minimal 10 µg, 400 ng µl^-1^) was extracted from pooled leaf powder from two biological samples for each treatment using the Spectrum^TM^ Plant Total RNA Kit (Sigma). RNA samples were subjected to DNase (Sigma) digestion to remove remaining DNA. mRNA was purified and enriched from total RNA samples by using poly-T oligo-coated magnetic beads (Invitrogen). Following purification and fragmentation, first-strand cDNA was generated using Superscript II reverse transcriptase (Invitrogen) and random hexamer primers. The cDNA was further converted into double stranded DNA using a strand-specific method according to Wang et al. [22]. After quality control of the cDNA libraries, pair-end sequencing analysis was carried out via Illumina HiSeq™ 2000 at the Beijing Genomics Institute (Shenzhen, China) according to the Illumina manufacturer’s protocol.

To ensure the accuracy of subsequent analysis, raw sequences were cleaned by removal of adaptors and sequencing errors. The reads containing the sequencing adaptor, more than 5% unknown nucleotides and more than 30% bases of quality value less than 10, were eliminated. This output was termed ‘clean reads’, which was used for alignment. All the clean reads were deposited in the National Center for Biotechnology Information (NCBI) and can be accessed in the Short Read Archive (SRA) linking to BioProject accession number: 196595. Prior to *de novo* assembly, the low-quality nucleotides at 3-ends were trimmed using a customer Perl script (CONDETRI: http://code.google.com/p/condetri) with the following parameters: hq=20, lq=10, frac=0.8, lfrac=0.1, minlen=40, mh=4, ml=1 and sc=64 [23]. To reduce the data complexity, two groups were generated from the clean reads from control (C_4_, C_10_ and C_13_) and fungal infected (I_4_, I_10_ and I_13_) samples for separate *de novo* assembly. The publicly available program Trinity (trinityrnaseq_r2012-05-18; http://trinityrnaseq.sourceforge.net/) was used for *de novo* transcriptome assembly of either group of trimmed clean reads to generate a set of contigs/transcripts with the following parameters: min_glue=2, SS_lib_type=RF, V=10, edge-thr=0.05, min_kmer_cov=3, path_reinforcement_distance=80 and group_pairs_distance=250 [24]. After the contigs from all samples were combined, the redundancy of contigs was removed and the contigs with partly overlap were elongated by the TGICL and Phrap assemblers [25]. The following parameters were used to ensure quality of assembly: a minimum of 95% identity between contigs, a minimum of 35 overlapping bases, a minimum of 35 scores and a maximum of 20 unmatched overhanging bases at sequence ends. Finally, based on the sequence similarity, the transcripts were divided into two classes: clusters (prefixed with ‘CL’) and singletons (prefixed with ‘unigene’). In each ‘CL’, the sequence similarity between transcripts was more than 70% and the transcripts were splice isoforms from one gene or paralogous gene.

### Identification of fungal transcripts, sequence annotation and bioinformatics analysis

To identify the fungal transcripts, we BLAST searched all transcripts in the S. *tritici* genome (http://genome.jgi-psf.org/Mycgr3/Mycgr3.home.html). More than 80% in sequence similarity with regard to the alignment length was required to be considered as a fungal transcript. BLAST search against the fungal genome using a threshold of 50% similarity was performed as well. Fungal proteins were assessed for signal peptides using SignalP (http://www.cbs.dtu.dk/services/SignalP). For annotation, wheat and fungal transcripts, respectively, were aligned to four public databases [non-redundant protein database (Nr) in NCBI, non-redundant nucleotide database (Nt) in NCBI, Swiss-Prot and Kyoto Encyclopedia of Genes and Genomes (KEGG) pathway database] by BLAST (E-value <10^-5^). Gene ontology (GO) classification was analysed by the Blast2GO software (v2.5.0) based on Nr annotation.

After annotation, the clean reads of each sample were mapped to all fungal and wheat transcripts using the SOAPaligner/soap2 software (BGI, China), allowing mismatches of no more than three bases, to obtain the transcripts from each sample. To minimize the influence of sequencing depth between samples, the total number of mapped reads was normalised by multiplying with a normalisation factor [26]. Expression level of the transcript was based on the number of unique match reads calculated and normalised to fragments per kilobase per million mapped fragments (FPKM) [27]. Analysis of differentially expressed wheat transcripts was performed as described by Chen et al. [28], which was based on the Poisson distribution [29] and normalisation for differences in sequencing depth between samples and gene length. A threshold of FPKM >2 in at least one sample, false discovery rate (FDR) <0.05 and at least two-fold change in FPKM of the transcript from infected *versus* control samples at minimum one time point was used to define differentially expressed wheat transcripts (performed by the in-house Perl script).

Principal component analysis (PCA) of sequencing data from all samples was performed by R script using log_2_FPKM values of transcripts in the samples. The expression profiles of the differentially expressed wheat transcripts [log_2_Ratio(infected/control)] and fungal transcripts (log_2_FPKM; FPKM>2 at minimum one time point) over the time course were determined by clustering analysis based on the k-means method using Euclidean distance. Heat map analysis of the differentially expressed wheat transcripts [log_2_Ratio(infected/control)] over the time course was performed using R script.

## Results

### Development of a bioassay to determine the disease transition

**Figure 1:**
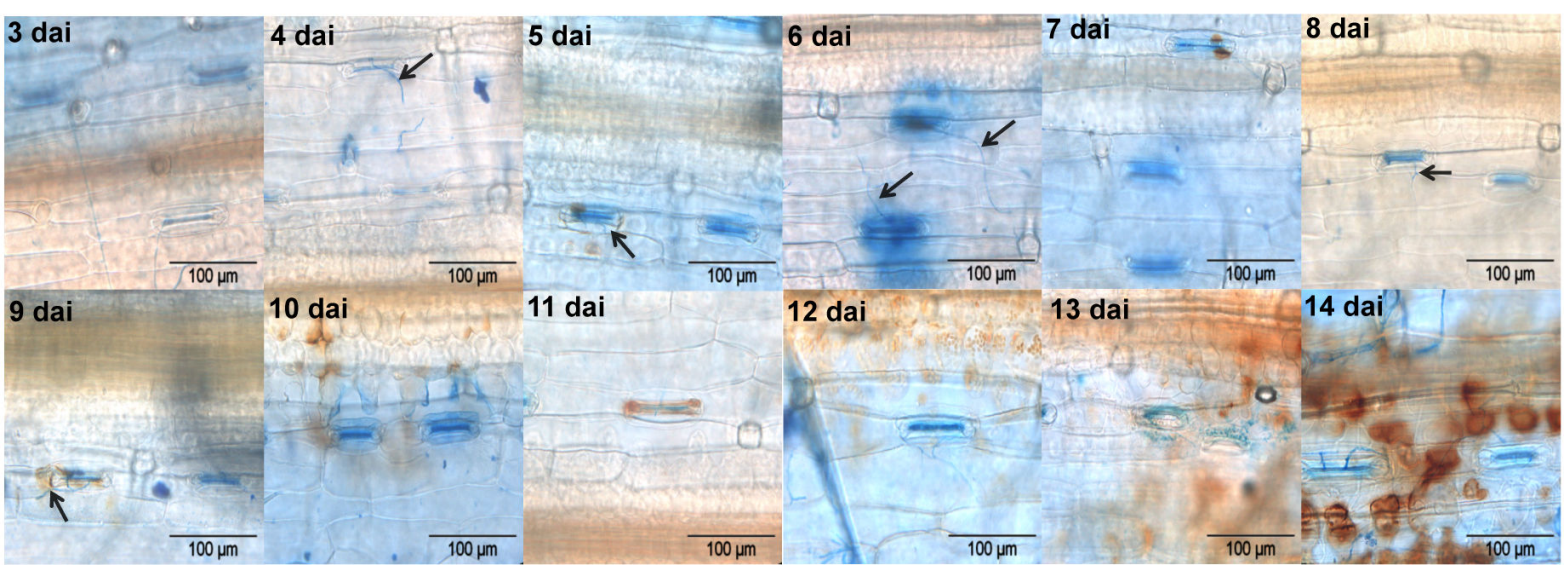
Detection of hydrogen peroxide in *S. tritici*-infected wheat leaves from 3 to 14 dai. DAB was used for staining H_2_O_2_ (red-brown staining). Fungal surface structures were stained with Evans blue. Arrows indicate attempted fungal penetration through the stomata.

**Figure 2:**
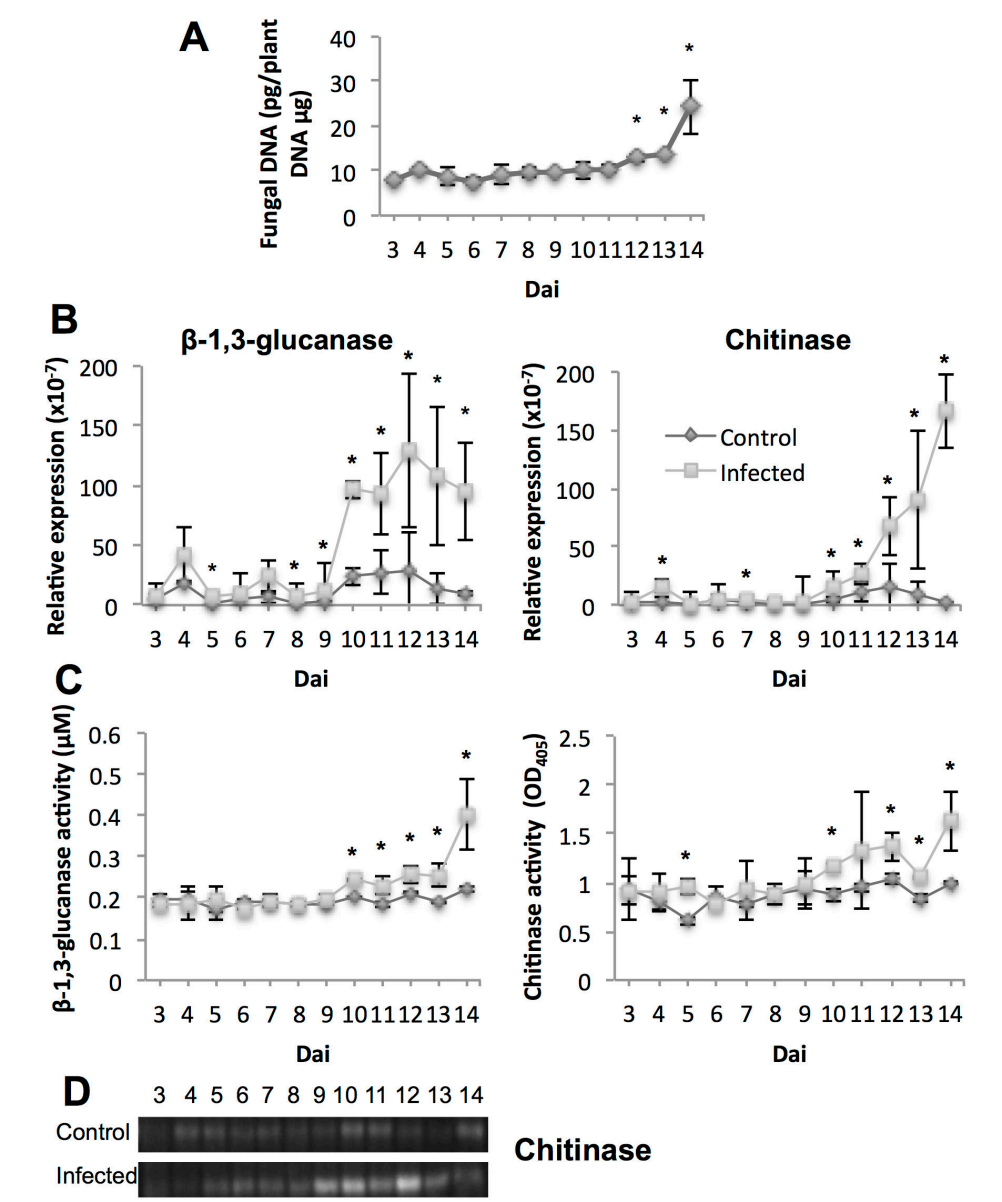
The bioassay to follow disease transition from 3 to 14 dai. (A) Fungal biomass expressed as content of *S. tritici* DNA. Significant increases (*P* < 0.05) in levels of fungal DNA are indicated by asterisks. (B) Wheat β-1,3-glucanase and chitinase gene expression analysed by qPCR. (C) β-1,3-glucanase and chitinase protein activities expressed as released glucose (μM) and OD_405_ measurement based on the assays, respectively. Significant differences (*P* < 0.05) between infected and control samples are indicated by asterisks. (D) Chitinase protein expression in protein extracts from control (upper panel) and *S. tritici*-infected leaves (lower panel) analysed by western blotting. Bands indicate chitinase. Representatives of two biological replicates at each time point are shown for the control and infected plants.

Given that the period of symptomless growth of *S. tritici* in wheat leaves can vary between experiments ranging from one week to several weeks and disease symptoms (Figure S1) are not uniform between leaves, we used the characteristic features of the biotrophic and necrotrophic phases in a bioassay to enable an interaction transcriptome analysis in the two distinct phases. The bioassay was based on characterisation of fungal biomass, H_2_O_2_ accumulation and plant PR-genes/proteins (β-1,4-glucanase and chitinase). The onset of a measurable increase in *S. tritici* growth has been shown to occur at day 9 and 10 [4,7]. Shetty et al. [3,15] additionally reported dramatically enhanced expression of β-1,3-glucanase and chitinase genes from 9 dai followed by extensive H_2_O_2_ accumulation, cell collapse and appearance of macroscopic symptoms from 12 dai during the compatible interaction. Therefore, we performed these assays over time in the compatible interaction to define the disease transition state. In the present study, fungal hyphae penetrated the stomata in the epidermis directly causing relatively little H_2_O_2_ accumulation up to day 8 (Figure 1). Increased accumulation of H_2_O_2_ in the substomatal cavities correlated with arrest of the progressing hypha was observed by day 9 and 10 (Figure 1), when strongly enhanced PR-gene expression and activity occurred in response to the fungus (Figure 2B–D). From 12 dai, the massive H_2_O_2_ accumulation in the mesophyll and hyphal development (Figure 1) were consistent with a significant increase in the levels of fungal biomass (Figure 2A). These results collectively show that following a long period of slow symptomless growth, the fungus switched to necrotrophy by day 9–10, advancing the increase in the biomass and massive H_2_O_2_ production by day 12. 

### A snapshot of transcript profiles during the wheat-*S. tritici* interaction

It is believed that the changes of lifestyle in a fungal pathogen and process of disease development are accompanied by stage-specific molecular events [30]. To gain comprehensive information on the molecular and physiological bases during particular stages of the interaction between wheat and *S. tritici*, we explored the complexity of transcriptional reprogramming of both host and pathogen at three time points (day 4, 10 and 13) by RNA-Seq analysis. The time points were selected based on the bioassay of marker genes/proteins and H_2_O_2_ accumulation and represented the initial symptomless phase, the intermediate transition stage and the necrotrophic phase, respectively. 

Table 1 summarises the sequencing data from *S. tritici*-infected wheat leaves and their respective controls. The mRNA sequencing workflow is shown in Figure S2. After the pre-processing of the reads, we obtained more than 12 million clean pair-end sequencing reads and 2G high-quality bases (Q20 bases approximately 97%) from both control samples and the mixed transcriptome samples (Table 1). In order to conduct dual sequencing analysis without the host reference genome, we used the *de novo* assembly method for control and infected samples separately. In total, 88770 non-redundant transcripts were obtained from all the samples including 12695 clusters (38680 transcripts) and 50090 singletons, the total length and N50 length of which were 63937118 bp and 1109 bp, respectively. Subsequently, we filtered the fungal transcripts by mapping all assembled transcripts to the fungal genome, resulting in identification of 1829 and 1811 fungal transcripts with a threshold of 50% and 80% sequence similarity, respectively (Table S1). In total, 313 fungal transcripts encoded proteins that were predicted to contain signal peptides. Functional categories were assigned to all annotated transcripts using GO classification and KEGG pathway analysis, resulting in 67610 and 1626 annotated wheat and fungal transcripts, respectively. Fungal (Table S1) and wheat (Tables S2, S3) transcripts with known function were grouped into 14 and 19 GO functional categories based on GO biological processes as well as 90 and 127 pathways, respectively. 

**Table 1:** Overview of sequencing transcriptome data from wheat leaves.

	C_4_	C_10_	C_13_	I_4_	I_10_	I_13_
Number of reads	12330423	12375478	12583655	12757779	12426764	13020331
Total base pairs (Mbp)	2220	2228	2265	2297	2237	2344
Q20 (%)	95.65	97.84	95.61	97.43	96.06	98.12
Total number of contigs	89315			59389		
Length of contigs (bp)	70551835			31332619		
N50 length of contigs (bp)	1233			692		
Number of mapped fungal transcripts	9	11	32	1578	1692	1822
Number of wheat transcripts	81976	79407	79642	79032	77927	75461
Highly expressed fungal transcripts	0	0	0	107	142	761
Highly expressed wheat transcripts	10158	11908	9563	9372	12715	13690

The transcripts with FPKM>10 are considered highly expressed. C_4_, control at 4 dai; C_10_, control at 10 dai; C_13_, control at 13 dai; I_4_, S*. tritici*-infected wheat at 4 dai; I_10_, S*. tritici*-infected wheat at 10 dai; I_13_, S*. tritici*-infected wheat at 13 dai.

**Figure 3:**
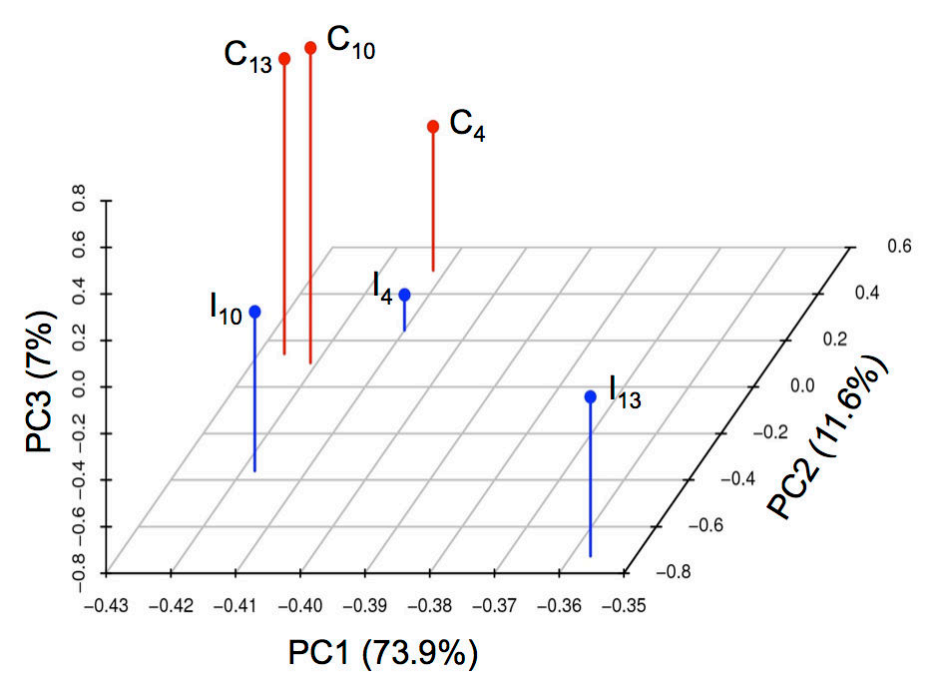
Principal component analysis of the transcriptome data. Principal components (PCs) 1, 2 and 3 account for 73.9, 11.6 and 7% of the variance, respectively. C_4_, control at 4 dai; C_10_, control at 10 dai; C_13_, control at 13 dai; I_4_, S. *tritici*-infected wheat at 4 dai; I_10_, S. *tritici*-infected wheat at 10 dai; I_13_, S. *tritici*-infected wheat at 13 dai.

Mapping the reads of each sample to wheat and fungal transcripts resulted in the identification of 1578, 1692 and 1822 fungal transcripts from infected samples at 4, 10 and 13 dai, respectively, as well as approximately 80000 non-redundant plant transcripts from each sample (Table 1). Nine, eleven and thirty-two low-expressed transcripts encoding tubulins, ATPases, ribosomal proteins and cytochrome b, respectively, were mapped as fungal transcripts from control samples, which is very likely due to the homology of these conserved genes between plant and the fungus. To further examine an overall relationship of the transcriptome data from different samples and effects of both fungal infection and interaction phases, PCA was conducted and clearly revealed that the greatest changes in host transcriptome were caused by the fungal infection following the interaction process (Figure 3). In particular, the 13 day-infected sample was separated from all other samples by principal component 1, accounting for 73.9% of the variance, strongly suggesting the accelerated host-pathogen interplay during the necrotrophic stage.

### Fungal transcriptome during the compatible interaction

**Figure 4:**
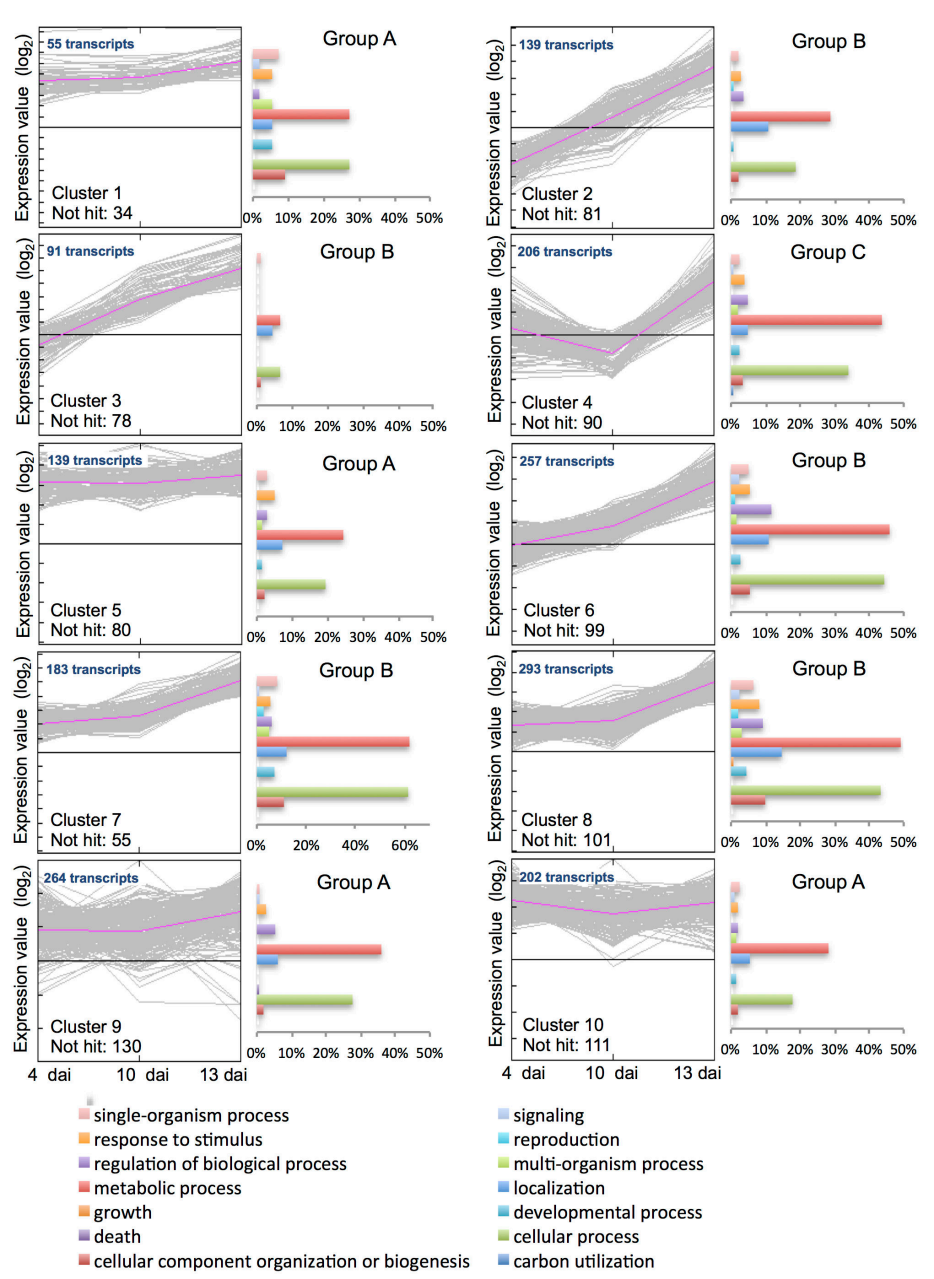
Functional category distribution in the ten expression clusters of fungal transcripts. The fungal transcripts were identified from *S. tritici*-infected wheat at 4, 10 and 13 dai. Expression level of the transcripts was calculated by log_2_FPKM. Histogram representation of the category distribution is expressed as percentage of the amount of transcripts belonging to the cluster. Transcripts coding for unknown products were included in the analysis. The clusters fall into three groups (A, B and C) based on the major profiling over the time course.

Figure 4 presents ten clusters of expression profiles of fungal transcripts as well as functional category distribution frequencies of the transcripts in each cluster. Not surprisingly, the transcripts with known function annotated in metabolic and cellular processes were dominating in each cluster. The clusters fell into three groups based on the major profiling over the time course: group A consisted of cluster 1, 5, 9 and 10, the majority of 660 transcripts showing constant abundance; group B consisted of cluster 2, 3, 6, 7 and 8, the majority of 963 transcripts showing increased abundance; group C consisted of cluster 4, the majority of 206 transcripts showing decreased abundance at 10 dai and highest abundance at 13 dai. A substantially higher number of transcripts annotated in signalling, responses to stimuli, reproduction, localisation, metabolic and cellular processes and cellular component organisation or biogenesis were represented in group B compared to other two groups (Figure 4). In addition, several transcripts involved in primary and energy metabolism and a considerable number of highly abundant transcripts annotated as ribosomal proteins were present in group B (Table S1). The FPKM values of housekeeping transcripts such as ribosomal and tubulin transcripts increased approximately 10-fold at 13 dai compared to the symptomless stage. These data suggest a highly active metabolic growth and an increase in fungal biomass at the destructive necrotrophic phase. 

Since the transcripts with the highest expression levels at specific growth stages likely play important roles in the control of the disease transition, we selected several transcripts with the highest expression levels at 4 dai (biotrophy) or 13 dai (necrotrophy) and listed them in Table 2. Besides the transcripts annotated as hypothetical proteins, most of which contained signal peptides and more than six cysteines, the transcripts were involved in pathogenesis and virulence, metabolism, signalling, transport, stress and defence, cell adhesion and cell wall degradation and remodelling (Table 2). Notably, these highly expressed transcripts at 4 and 13 dai precisely belonged to group A and B, respectively, indicating that the transition of *S. tritici* lifestyle was likely accompanied and regulated by the expression of stage-specific sets of genes. More interestingly, although seven transcripts involved in host cell wall degradation including glycoside hydrolase families 5, 16 and 76 in group A and glycoside hydrolase families 3 and clan CH-D in group C were identified, none of them were of high abundance at either stage (FPKM <8; Table S1). 

**Table 2:** Expression patterns of selected *S. tritici* transcripts during infection.

		**FPKM**					
**ID**	**Annotation**	**4 d**	**10 d**	**13 d**	**Biological process**	**S**	**C**
**Transcripts highly expressed (FPKM ≥ 8) at 4 d**							
96543	Hydrophobin 2	9.9	23.1	15.6	Pathogenesis and virulence	Yes	5
70312	Peptidase S8 and S53	10.2	12.4	23.3	Metabolism	Yes	5
74298	Glucoamylase	15.9	16.0	19.0	Metabolism	Yes	5
77672	Esterase/lipase/thioesterase	19.5	15.8	8.8	Metabolism	No	5
81692	β-1,6-N-acetylglucosaminyltransferase	13.2	5.7	6.9	Metabolism	Yes	5
104538	β-1,6-N-acetylglucosaminyltransferase	52.0	44.5	47.8	Metabolism	No	1
86867	NADPH-dependent FMN reductase	33.7	36.0	102.0	Metabolism	Yes	1
100165	Fumarate lyase	39.3	11.8	32.3	Metabolism	No	1
108219	Autophagy-related protein 8	11.9	9.9	28.6	Metabolism	No	5
109517	Carbohydrate kinase, PfkB	17.0	6.4	10.1	Metabolism	No	5
102849	Homeobox transcription factor prospero	14.2	11.2	37.2	Metabolism	Yes	1
44262	Thiamine biosynthesis Thi4 protein	11.0	6.9	13.0	Metabolism	No	5
104937	Pyridoxamine 5'-phosphate oxidase	17.8	5.3	14.2	Metabolism	No	5
104409	Similar to bacterial rhodopsin	29.3	40.3	70.2	Signalling	No	1
109991 93710	Serine/threonine protein kinase	62.4	57.3	247.0	Signalling	Yes	1
99917	Serine/threonine protein kinase	19.9	12.9	16.9	Signalling	Yes	5
97868	Tyrosine protein kinase	16.6	25.4	72.0	Signalling	Yes	1
110667	PAS-domain protein	63.8	70.2	222.0	Signalling	Yes	1
39898	Aquaporin	24.9	10.8	11.8	Transport	No	5
42411	GPR1/FUN34/yaaH membrane protein	20.9	20.1	12.4	Transport	No	5
58200	ABC transporter	11.1	6.8	10.2	Transport	No	5
76071	Phosphate transporter	19.9	13.6	12.3	Transport	Yes	5
105313	Sugar transporter superfamily	20.8	12.9	12.4	Transport	No	5
64952	Cyclophilin	10.5	11.9	40.6	Stress and defence	No	1
72265	Cyclophilin	12.2	4.5	11.0	Stress and defence	No	5
106153	Carbohydrate-binding WSC	18.2	19.1	25.2	Stress and defence	No	1
59219	Haem peroxidase	9.7	5.7	2.7	Stress and defence	No	10
94368	Chloroperoxidase	10.9	14.5	6.7	Stress and defence	Yes	5
101235	Chloroperoxidase	12.1	16.5	14.8	Stress and defence	Yes	5
103393	Allergen V5/Tpx-1 related	23.1	24.8	63.8	Stress and defence	Yes	1
103593	Cu^2+^/Zn^2+^ superoxide dismutase 1	12.8	20.1	57.3	Stress and defence	No	1
105791	Aldehyde dehydrogenase	28.6	19.4	20.4	Stress and defence	No	1
101210	Zinc-containing alcohol dehydrogenase	9.6	2.3	4.3	Stress and defence	No	10
56432	Fasciclin	9.1	1.8	5.2	Cell adhesion	Yes	10
104730	von Willebrand factor	24.4	21.6	31.4	Cell adhesion	Yes	1
100647	Chitinase	9.6	1.4	9.4	Cell wall degradation and remodelling	Yes	10
106219	Glycolipid anchored surface protein GAS1	12.4	3.3	21.3	Cell wall degradation and remodelling	Yes	5
90001	Yeast PIR protein repeat-like	38.4	36.7	73.6	Cell wall degradation and remodelling	Yes	1
69789	Hypothetical protein	21.6	9.9	19	--	Yes	5
94290	Hypothetical protein	112	88.1	54.4	--	Yes	1
106329	Hypothetical protein	25.4	22.5	9.5	--	Yes	5
103950	Hypothetical protein	14.7	7.6	26.1	--	Yes	5
111606	Hypothetical protein	8.2	10.0	14.3	--	Yes	5
**Transcripts highly expressed (FPKM ≥ 10) at 13 d**							
39947	Cerato-platanin	1.2	2.9	44.3	Host immune system	Yes	3
105487	Peptidoglycan-binding LysM	1.2	12.7	119.8	Pathogenesis and virulence	Yes	3
111221	Peptidoglycan-binding LysM	3.0	12.1	105.3	Pathogenesis and virulence	Yes	7
88451	Necrosis-inducing protein NPP1	0.7	3.5	10.5	Pathogenesis and virulence		
104853	Ubiquinol-cytochrome C reductase	4.0	4.5	23.5	Metabolism	Yes	7
107092	β-1,6-N-acetylglucosaminyltransferase	2.6	2.4	20.9	Metabolism	Yes	8
108724	Cysteine peptidase	3.3	3.9	29.1	Metabolism	No	7
95071	Nucleoside diphosphate kinase	5.1	3.4	33.3	Metabolism	No	7
57734	G-protein beta WD-40 repeat	3.1	1.3	23.9	Signalling	No	8
86648	G-protein beta WD-40 repeat	2.9	4.9	22.1	Signalling	No	7
69026	Translationally controlled tumor protein	5.7	7.3	31.9	Signalling	No	7
78859	EGF-like	0.9	2.5	34.4	Signalling	No	3
103900	EGF-like	0.2	21.2	120.3	Signalling	Yes	3
106452	Serine/threonine protein kinase	3.5	11.8	71.2	Signalling	Yes	7
100205	Phox-like	5.5	5.2	24.2	Signalling	No	7
65946	Amino acid permease	0.6	5.3	29.7	Transport	No	3
73144	Ammonium transporter	0.1	7.1	24.4	Transport	No	3
77435	Porin, eukaryotic type	6.0	3.1	26.8	Transport	No	7
65963	Cytochrome b561	0.1	0.7	26.6	Transport	Yes	2
90089	Ankyrin	0.1	7.0	42.7	Transport	Yes	3
82936	Plastocyanin-like	4.9	6.3	32.8	Transport	Yes	7
98411	Cytochrome P450	0.4	3.4	20.6	Stress and defence	No	3
55916	Glutathione S-transferase	3.4	2.5	20.6	Stress and defence	No	8
71387	Alkyl hydroperoxide reductase	3.6	3.6	29.4	Stress and defence	No	7
101587	Thioredoxin	2.2	5.0	41.4	Stress and defence	Yes	7
104975	Peroxiredoxin	3.4	4.6	26.3	Stress and defence	No	7
72449	Heat shock protein Hsp70	2.7	6.2	23.3	Stress and defence	No	7
83835	Heat shock protein Hsp70	1.6	3.3	24.3	Stress and defence	Yes	8
103427	von Willebrand factor	4.5	12.8	141.1	Cell adhesion	Yes	7
106780	von Willebrand factor	2.6	3.3	36.7	Cell adhesion	Yes	7
102341	Glycolipid anchored surface protein GAS1	7.3	4.7	28.3	Cell wall degradation and remodelling	Yes	7
102481	Yeast PIR protein repeat-like	0.7	2.3	43.1	Cell wall degradation and remodelling	Yes	3
104794	Chitinase	0.4	2.0	36.0	Cell wall degradation and remodelling	Yes	3
44596	Kinesin, motor region	4.1	21.5	40.4	Locomotion	No	7
83064	Hypothetical protein	0.6	3.4	56.2	--	Yes	3
79161	Hypothetical protein	1.4	3.8	36.8	--	Yes	3
89705	Hypothetical protein	0	0	23.0	--	Yes	7
92097	Hypothetical protein	0.7	6.7	24.3	--	Yes	3
97031	Hypothetical protein	0.6	3.6	22.4	--	Yes	3
110220	Hypothetical protein	2.4	9.8	68.2	--	Yes	7
102792	Hypothetical protein	5.4	63.7	277.0	--	Yes	1
104404	Hypothetical protein	0.4	12.2	60.0	--	Yes	3
104444	Hypothetical protein	1.0	24.9	102.7	--	Yes	3
105825	Hypothetical protein	0.8	14.9	74.9	--	Yes	3
105826	Hypothetical protein	1.5	9.7	35.7	--	Yes	3
108482	Hypothetical protein	0.1	0.3	20.7	--	Yes	2
107904	Hypothetical protein	0.3	3.7	43.8	--	Yes	3
107286	Hypothetical protein	0.9	3.3	29.8	--	Yes	3

FPKM indicates the expression level of the transcript. Most of the hypothetical proteins are small cysteine-rich proteins.

S, SignalP; C, cluster number in Figure 4; d, days after inoculation.

Furthermore, since fungal secreted proteins may have potential roles as effectors, 313 transcripts encoding proteins containing signal peptides, the majority of which were hypothetical proteins, peptidases, glycoside hydrolases, antioxidants, kinases, heat shock proteins and oxidoreductases, were clustered to obtain the expression patterns (Figure S3A). Their expression levels increased (clusters 1, 3, 4, 7–10) or remained constant (clusters 2, 5 and 6) over the time course. Likewise, of 492 candidate virulence effectors previously predicted *in silico* [12], 246 fungal transcripts were identified in the present study with increased or constant expression levels during infection (Figure S3B; Table S1). 

### Wheat leaf transcriptome in response to infection

In total, 33314 wheat transcripts, fulfilling the selection criteria, were identified in response to the pathogen (Table S2). Cluster analysis and category distribution were performed to assess the overall patterns of host transcriptome changes over the time course of infection (Figure S4). Clusters 1 and 3 contained 3266 transcripts mainly regulated by the fungus at 4 dai. Clusters 5, 6, 7 and 9 contained 16543 transcripts mainly regulated at 13 dai. Clusters 2, 4 and 8 contained 11324 transcripts mainly regulated at 10 dai. Additionally, cluster 10 contained 2181 transcripts down-regulated and up-regulated at 4 and 13 dai, respectively. The major transcripts in each cluster were annotated in signalling, response to stimuli, reproduction, regulation of biological process, metabolic and cellular processes, locomotion, localisation, development, cellular component organisation or biogenesis and biological adhesion. Considerably higher numbers of transcripts involved in metabolism, transport, defence and stress, signalling and cellular component organisation changed in abundance at 10 and 13 dai (Figure S4; Table S2), suggesting a strong induction of metabolic and defence-activities from 10 dai, which was associated with increased H_2_O_2_ production (Figure 1). 

**Figure 5:**
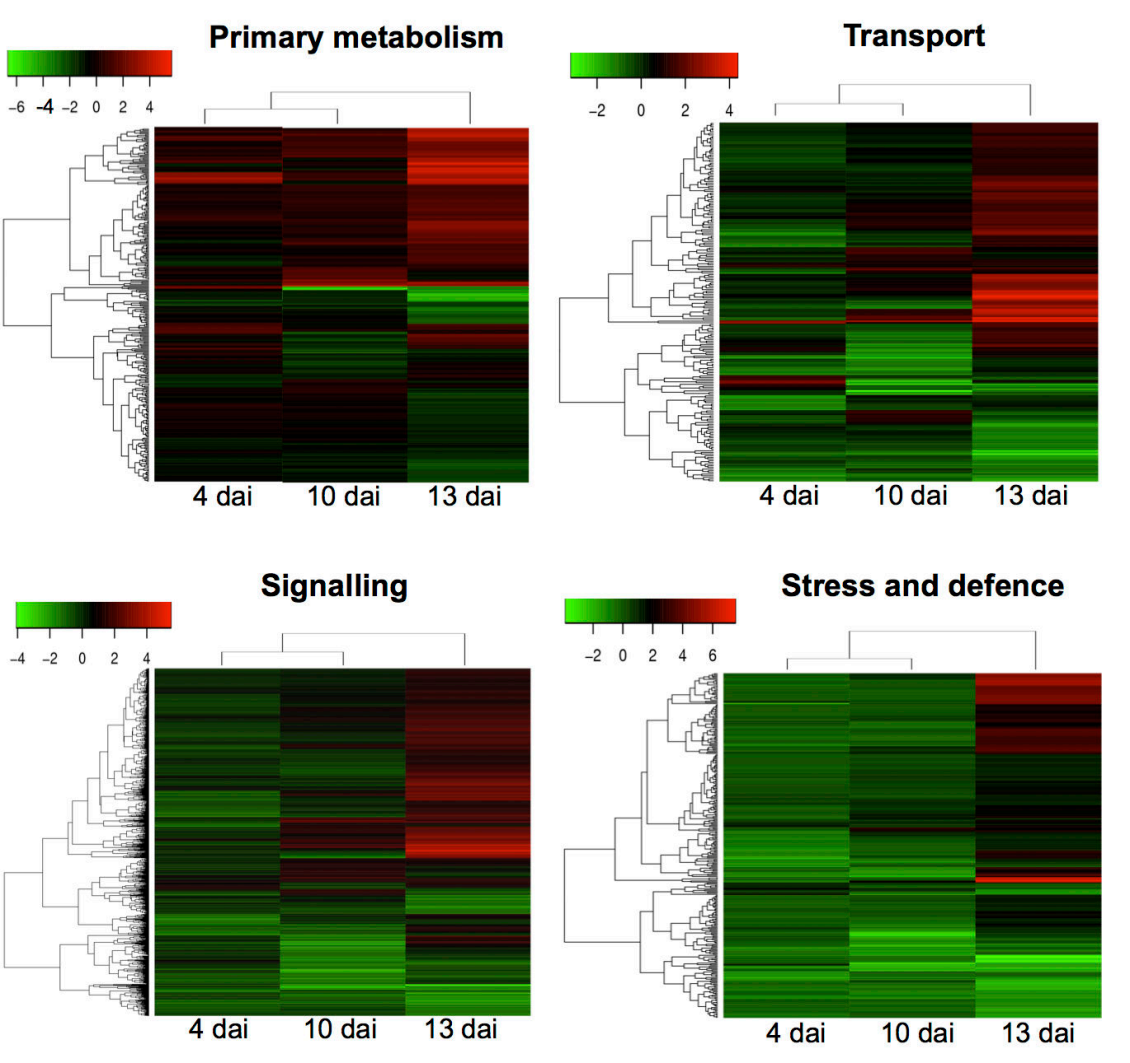
Heat map visualization of differentially expressed wheat transcripts. The plant transcripts are mainly involved in primary metabolism, signalling, transport and stress and defence in response to *S. tritici*. Differential expression patterns are based on the log_2_ fold changes of transcript abundance in *S. tritici*-infected wheat *versus* controls at 4, 10 and 13 dai.

To gain further insights into the responses of specific functions of host genes in fungal infection at different stages, we carried out a heat map analysis of the transcripts. This included genes specifically annotated in primary metabolism (e.g., metabolism of carbohydrates, nitrogen and amino acids as well as photosynthesis), transport (e.g., ATPases, ABC transporters, aquaporins and sugar, amino acid, peptide, nucleic acid and lipid transporters) and signalling [e.g., membrane-bond receptors, receptor kinases, G proteins, phospholipids, calcium-dependent protein kinases, MAPK pathways, WRKYs and other transcription factors, hormone pathways (salicylic acid, jasmonate, ethylene, auxin, gibberellic acid and brassinosteroid)] as well as genes involved in stress and defence (e.g., oxidative stress, detoxification, PR-genes, heat shock proteins, the phenylpropanoid pathway, LRR domain proteins and other stress-related genes) (Figure 5). The analysis revealed two distinct gene expression patterns of the host in response to the fungus during the biotrophic and necrotrophic stages. At the early symptomless stage, the fungus caused minor alterations in the expression of host transcripts involved in primary metabolism, transport and signalling, although some transcripts significantly changing in abundance were observed. By contrast, a considerable number of transcripts involved in defence and defence-related signalling, encoding proteins such as antioxidants, PR-proteins, heat shock proteins, disease resistance proteins, WRKY transcription factors and NAC domain proteins were slightly down-regulated at this stage, even though some, such as receptor-like kinases and phytohormone-inducing proteins, could be up-regulated (Figure 5; Table S2). At the necrotrophic stage, great changes were observed in expression of host transcripts involved in host metabolism, transport, signalling and defence with up-regulation of a substantial number of transcripts (Figure 5). Furthermore, it is worth to note that levels of the major transcripts annotated in carbohydrate metabolism were up-regulated at this stage, whereas major transcripts annotated in photosynthesis were down-regulated. Additionally, several transcripts involved in anti-oxidative stress were strongly down-regulated at the late necrotrophic stage (Table S2), which was correlated to massive accumulation of H_2_O_2_ (Figure 1). 

## Discussion

Understanding the molecular mechanisms underlying fungal infection and host plant defence is a prerequisite for understanding the host-pathogen interaction and can contribute to development of new strategies of crop protection against pathogen. A global study of host and fungal transcriptional reprogramming during the interaction allows us to gain insights into such mechanisms. Here, we report the first use of RNA-Seq technique in the compatible interaction between wheat and *S. tritici*, with particular focus on the metabolic and physiological alterations of both host and pathogen during the shift from biotrophic to necrotrophic growth stages. 

It has been suggested that the transition from slow biotrophic growth to rapid necrotrophic growth is associated with distinct expression patterns of genes regulated by nutrient status, revealed by comparison of transcriptomes between *in planta* transition/necrotrophic stage and *in vitro* nutrition-rich/limited conditions [4,13]. Our interaction transcriptome analysis directly demonstrates two different phases of the compatible interaction: i) slow symptomless growth associated with minor host molecular responses and ii) necrotrophic feeding/high metabolic growth associated with enhanced metabolic and defence responses in plant. The symptomless growth of *S. tritici* was achieved by suppressing or attenuating host defence responses at transcription level (Figure 5), despite up-regulation of some defence-related genes such as receptor kinases, phytohormone-inducing proteins and PR-2 and 3 as well as accumulation of stress-related compounds such as ROS. This symptomless growth is analogous with most biotroph lifestyles of both repression of plant defence responses and induction of host specific genes. However, accumulation of several defence-related proteins and down-regulation of several proteins involved in photosynthesis have been observed at the biotrophic stage as revealed by proteomics [16]. Down-regulation of several defence-related transcripts and no alteration in the levels of several photosynthesis-related transcripts appear not to be correlated to the regulation behaviour of their protein products. The poor correlation between transcript and protein levels has been well demonstrated due to the regulation of transcription, RNA processing, translation and protein modifications and turnover and furthermore suggests that integrated ‘omics’ studies are required to comprehensively understand the molecular processes in the biological system. On the other hand, the necrotrophic growth of *S. tritici* triggers the plant cells to create a highly defensive and metabolic active environment, generating ROS and strongly inducing defence, signalling and metabolism-related genes. The crosstalk between induced signalling events, including receptor-mediated signal perception, protein phosphorylation, ion fluxes, production of ROS, generation and regulation of secondary signalling molecules (e.g., phytohormones and WRKYs), leading to the activation of defence genes, has been well established as the mechanisms of host plant defence to different pathogens [31]. More interestingly, the highly oxidative cell environment in wheat during the necrotrophic stage was accompanied by down-regulation of large number of plant transcripts encoding ROS-scavenging enzymes, thus allowing the ROS to accumulate. For some necrotrophic pathogens, it has been suggested that ROS accumulation and depletion of host antioxidants can result in increased susceptibility as well as host necrosis [32]. Interestingly, this is not the case for *S. tritici* that is able to tolerate the high amounts of H_2_O_2_ accumulating during the necrotrophic growth phase, but does not thrive in this environment since scavenging of H_2_O_2_ boosts fungal growth [3,7]. Furthermore, levels of transcripts involved in photosynthesis were suppressed during the necrotrophic phase, whereas carbohydrate and energy metabolism was up-regulated in the host and associated with enhanced defence responses [7]. It has been demonstrated that the activation of plant defence responses is cost-intensive and causes a decrease in photosynthesis and an increased demand for assimilates, energy, reducing equivalents and carbon skeleton components that are provided by the primary metabolic pathways [33]. Simultaneously, the pathogen will put pressure on the host carbohydrate metabolism for its own needs, which will further increase the demand for assimilates. 

To date, little is known of the molecular mechanisms in *S. tritici* that promote an early symptomless, biotrophic phase and a late highly destructive necrotrophic stage or those that mediate the transition between the two. Previous investigations focused on the secreted protein effectors for facilitating the initial symptomless growth and triggering PCD [10]. We observed strong, differential and growth stage-specific expression of transcripts encoding several small secreted cysteine-rich proteins with unknown function at the two stages, a hydrophobin 2 during the biotrophic stage and two LysM proteins, a necrosis-inducing protein and a cerato-platanin during the necrotrophic stage, which may have potential roles in pathogenesis or suppressing plant defence. This observation suggests that *S. tritici* secretes distinct classes of proteins that may first suppress plant defence responses and later induce host necrosis. It has been reported that some fungal effectors, for instance, *P. infestans* Avr3a and SNE1 and *P. sojae* Avr1b, can suppress PCD or host defences during biotrophic stages [34–36]. On the other hand, the P. *infestans PiNPP1.1* gene encoding a NLP effector, which is only highly expressed during necrotrophy, induces necrosis and is suppressed by the SNE1 effector [37]. Both LysM proteins identified in the present study have been functionally characterised, one of which was proposed to be an effector to facilitate the symptomless leaf colonisation of *S. tritici* through its activity in suppressing chitin-mediated plant defences [8]. Surprisingly, LysM transcripts were highly expressed during the necrotrophic stage here, which was associated with increased fungal chitin/biomass and plant defence responses. Thus, our data suggest that *S. tritici* manipulates the suppression of chitin-mediated wheat defences during the entire infection process and may be important for disease development due to the increased level of biomass or chitin. Expression of a necrosis-inducing protein has also been found in the oomycete Phytophthora *sojae* during transition from biotrophy to necrotrophy in soybean, which suggests that this protein facilitates the plant colonisation during the necrotrophic growth [38]. Three secreted necrosis-inducing protein effectors have been identified in barley pathogen *Rhynchosporium commune* and shown their contribution to disease development [39]. Cerato-platanin in phytopathogenic fungi acts as a phytotoxin and a pathogen-associated molecular pattern (PAMP) due to the induction of defence responses including phytoalexin synthesis and plant cell death [40]. Their strong expression at the transition and the necrotrophic stages, which was associated with the enhanced host defence responses, indicates a potential role in induction of the disease transition and/or facilitation of the necrotrophic growth. Fungal secreted small cysteine-rich hydrophobins have been implied in pathogenesis through acting as toxins or attaching fungal structures to the host surface [41]. Eight hydrophobin-like proteins are predicted in the annotated *S. tritici* genome, four of which are secreted [12]. In addition to one highly expressed hydrophobin shown in Table 2, four additional hydrophobins were identified with one containing a signal peptide here (Table S1). We speculate that hydrophobins play a role in facilitating the initial symptomless growth through its ability of mediating fungal hypha to attack the host cells. 

The coverage of the transcripts encoding the proteins involved in transport, signalling and stress responses among the identified fungal transcripts was remarkable at both stages, indicating their important roles in facilitating both biotrophic and necrotrophic growth. Strongly expressed transcripts involved in defence-related signalling included G proteins, serine/threonine protein kinases (likely involved in MAPK pathways or Ca^2+^ signalling), PAS-domain proteins functioning as sensors of oxygen, redox potential, light and other stimuli [42] and EGF-like proteins known for a role in the immune system [43]. Some of these identifications differed from the 74 transcripts involved in four MAPK pathways, the cAMP-dependent pathways and G-signalling identified in a previous *in planta* EST sequencing study [5]. Disruption of *S. tritici* signalling genes can significantly affect and regulate the invasive growth, penetration or pathogenicity [44]. With respect to highly expressed transport-related transcripts, different classes corresponding to two growth stages were identified. The nutrient uptake and transport facilitation appeared to be active particularly during the biotrophic stage in contrast to the primary metabolism-related transport during the necrotrophic growth. As described above, ROS accumulated in the host from 3 to 14 dai (Figure 1) and is considered as one of the primary responses to *S. tritici*, starting at the infection site during the biotrophic stage and spreading to the entire tissue at the late necrotrophic stage [3]. ROS are effective in stopping growth of biotrophic pathogens. However, the roles of ROS in interactions with necrotrophic fungi are still ambiguous. It can both benefit and inhibit infection by necrotrophs, which may be able to produce ROS themselves and stimulate the ROS production from the hosts as well [7,45]. To survive in the harsh oxidative environments and colonise host cells, *S. tritici*, therefore, has to develop mechanisms to scavenge ROS and protect against ROS-induced damage, particularly during the necrotrophic stage when massive ROS accumulation occurs [3]. This was evident in the present study by increased expression levels of several fungal transcripts during infection encoding ROS-scavenging proteins including peroxidases, Cu^2+^/Zn^2+^ superoxide dismutases, glutathione S-transferases, hydroperoxide reductases, thioredoxins and peroxiredoxins as well as other stress-response proteins like cyclophilins and heat shock proteins (Table S1). Furthermore, excessive ROS production in the fungal cells after expose to the stress can give rise to the accumulation of aldehydes and alcohols [46]. *S. tritici* may rescue itself by increased expression of aldehyde dehydrogenase and alcohol dehydrogenase for detoxification of alcohols and aldehydes (Table 2). 

During the entire infection cycle, *S. tritici* grows intercellularly throughout the leaf mesophyll cell layer and the host cell walls appear not to be breached [2,3], although the fungus can express a series of genes encoding CWDEs *in planta*, in particular a large number during the very late necrotrophic stage when the foliar tissue is fully necrotic, reflecting that stage of pathogenesis [4,5]. CWDEs are known to play a role in nutrient acquisition, host colonisation and pathogenicity in phytopathogenic fungi. However, scanning the S. *tritici* genome indeed revealed a relatively small number of genes annotated as CWDEs compared to some saprophytes and phytopathogenic fungi like *Fusarium graminearum*, *Magnaporthe oryzae* and *Stagonospora nodorum* [9,12]. Little is understood about the biological explanations for the strictly apoplastic colonising hyphae of *S. tritici* which do not breach host cell walls. In the present study, we identified relatively few transcripts encoding CWDEs expressed during the biotrophic and initial necrotrophic stages out of the total identifications and none of them was of high abundance, indicating that degradation of host cell walls may not play an essential role for nutrient acquisition and explaining the reduced capacity for breaching living host cell walls. Correspondingly, Rohel et al. [47] found that during the transition to necrotrophic growth where sporulation started, the fungus actually starved, indicating deficiency in obtaining sufficient nutrients. However, the fungus has to survive on the nutrients in the apoplast full of proteins, metabolites, sugars and ions [47]. Here, the identification of several transcripts annotated as peptidases, lipases and amylases with high abundance at two stages (Table 1, S2) suggests that degradation of host cell proteins, lipids and sugars can be an important source of fungal nutrients during colonisation. This is in agreement with the genomic mapping analysis revealing that several protease encoding gene families are expanded and 39 proteins have functions related to protein degradation [12].

In summary, the disease transition was clearly defined by characterising accumulation of H_2_O_2_, PR-proteins and fungal biomass in order to carry out the transcriptome analysis at the biotrophic and necrotrophic stages of the compatible interaction between wheat and *S. tritici*. RNA-Seq enabled the identification of considerable numbers of fungal and wheat transcripts in response to the infection at both stages. The results suggest that successful leaf colonisation is achieved by suppressing host defence responses during the symptomless biotrophic stage, activation of signalling, transport and antioxidative mechanisms, apoplastic nutrient acquisition and secretion of distinct classes of stage-specific molecules including potential effectors. The necrotrophic growth triggers enhanced host responses including anti-stress and defences, energy metabolism, signalling and transport as well as decreased photosynthesis. This transcriptome study paves the way for more detailed work on *S. tritici* pathogenicity and wheat resistance to *S. tritici*, which will be important for future crop protection. 

## Supporting Information

Figure S1 **Macroscopic appearance of susceptible wheat leaves after treatment with water (controls) or** ***S. tritici***. The leaves were photographed from 3 to 14 dai.
(TIF)

Figure S2 **Schematic representation of RNA-Seq analysis of the interaction transcriptome between wheat and *S. tritici*.** Total RNA was extracted from control and *S. tritici*-infected samples at 4, 10 and 13 dai. Illumina RNA-Seq was performed on cDNA libraries generated from each sample. All pre-processed reads of controls as well as the infected samples were combined and assembled. Subsequently, the transcripts from all the samples were combined, mapped to the fungal genome to filter out fungal transcripts prior to annotation. Finally, the clean reads of each sample were mapped to wheat and fungal transcripts. Bioinformatics analysis was conducted to obtain expression levels of both wheat and fungal transcripts in each sample and define differentially expressed wheat transcripts at each time point.
(TIF)

Figure S3 **Expression profiles of *S. tritici* transcripts encoding potential effector proteins identified from *S. tritici*-infected wheat at 4, 10 and 13 dai.** Expression levels of the transcripts were calculated by log_2_FPKM. (A) Ten expression clusters of 313 fungal transcripts encoding proteins containing signal peptides. (B) Ten expression clusters of 246 fungal transcripts which have been identified in the present study and *in* *silico* predicted as effectors by do Amaral et al. [12].
(TIF)

Figure S4 **Functional category distribution in the ten expression clusters of differentially expressed wheat transcripts.** Differentially expressed plant transcripts in response to *S. tritici* at 4, 10 and 13 dai are presented by expression ratios of the transcripts in infected (I) *versus* control (C) samples in log_2_-scale. Histogram representation of the category distribution is expressed as percentage of the amount of transcripts belonging to the cluster. Transcripts coding for unknown products were included in the analysis.
(TIFF)

Table S1 **List of identified *S. tritici* transcripts during infection of wheat leaves at 4, 10 and 13 dai.**
(XLSX)

Table S2 **List of differentially expressed wheat transcripts in response to *S. tritici* at 4, 10 and 13 dai.**
(XLSB)

Table S3 **List of identified wheat transcripts with no change in abundance during *S. tritici* infection at 4, 10 and 13 dai.**
(XLSB)
