# Supplementary figures and images for: Transcriptional Reprogramming of Wheat and the Hemibiotrophic Pathogen Septoria tritici during Two Phases of the Compatible Interaction
Source: PLoS One. 2013 Nov 26;8(11):e81606. doi: 10.1371/journal.pone.0081606 (PMC3841193; doi:10.1371/journal.pone.0081606)

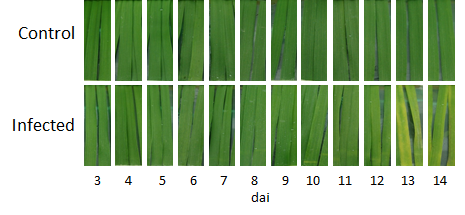

Supplement: Figure S1 — Macroscopic appearance of susceptible wheat leaves after treatment with water (controls) or S. tritici. The leaves were photographed from 3 to 14 dai. (TIF) [file pone.0081606.s001.tif]

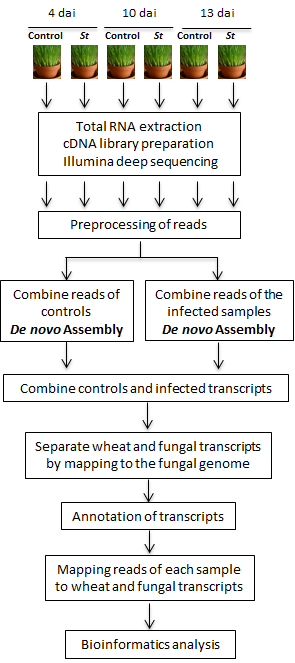

Supplement: Figure S2 — Schematic representation of RNA-Seq analysis of the interaction transcriptome between wheat and S. tritici. Total RNA was extracted from control and S. tritici-infected samples at 4, 10 and 13 dai. Illumina RNA-Seq was performed on cDNA libraries generated from each sample. All pre-processed reads of controls as well as the infected samples were combined and assembled. Subsequently, the transcripts from all the samples were combined, mapped to the fungal genome to filter out fungal transcripts prior to annotation. Finally, the clean reads of each sample were mapped to wheat and fungal transcripts. Bioinformatics analysis was conducted to obtain expression levels of both wheat and fungal transcripts in each sample and define differentially expressed wheat transcripts at each time point. (TIF) [file pone.0081606.s002.tif]

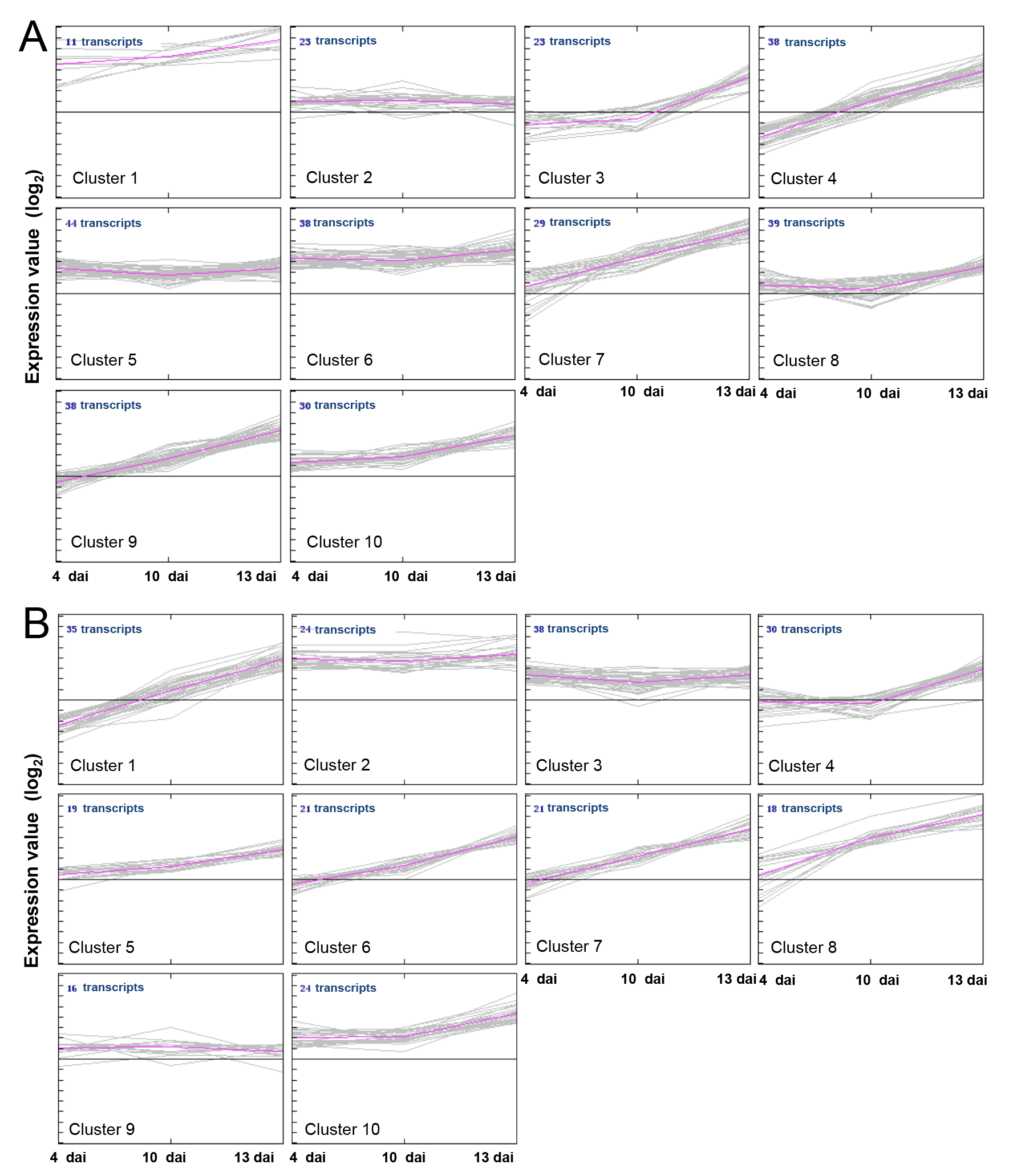

Supplement: Figure S3 — Expression profiles of S. tritici transcripts encoding potential effector proteins identified from S. tritici-infected wheat at 4, 10 and 13 dai. Expression levels of the transcripts were calculated by log2FPKM. (A) Ten expression clusters of 313 fungal transcripts encoding proteins containing signal peptides. (B) Ten expression clusters of 246 fungal transcripts which have been identified in the present study and in silico predicted as effectors by do Amaral et al. [12]. (TIF) [file pone.0081606.s003.tif]

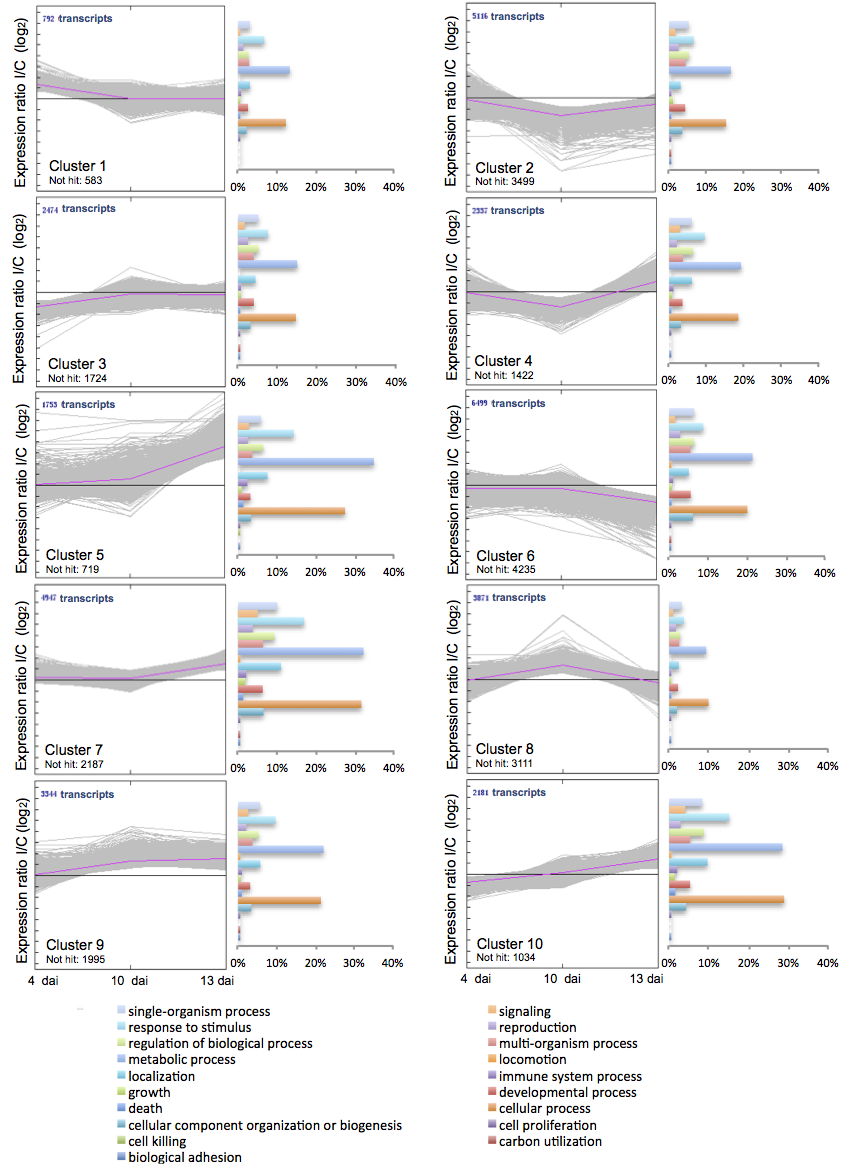

Supplement: Figure S4 — Functional category distribution in the ten expression clusters of differentially expressed wheat transcripts. Differentially expressed plant transcripts in response to S. tritici at 4, 10 and 13 dai are presented by expression ratios of the transcripts in infected (I) versus control (C) samples in log2-scale. Histogram representation of the category distribution is expressed as percentage of the amount of transcripts belonging to the cluster. Transcripts coding for unknown products were included in the analysis. (TIFF) [file pone.0081606.s004.tiff]
